# Atopic dermatitis

**DOI:** 10.1186/s13223-018-0281-6

**Published:** 2018-09-12

**Authors:** Sandeep Kapur, Wade Watson, Stuart Carr

**Affiliations:** 1 IWK Health Centre, Division of Allergy, Department of Pediatrics, Dalhousie University, Halifax, NS Canada; 2 Department of Pediatrics, University of Alberta, Edmonton, AB Canada

**Keywords:** Atopic dermatitis, Diagnosis and management, Emollients, Skin care practices, Topical corticosteroids, Topical calcineurin inhibitors

## Abstract

Atopic dermatitis (AD) is a common, chronic skin disorder that can significantly impact the quality of life of affected individuals as well as their families. Although the pathogenesis of the disorder is not completely understood, it appears to result from the complex interplay between defects in skin barrier function, environmental and infectious agents, and immune dysregulation. There are no diagnostic tests for AD; therefore, the diagnosis is based on specific clinical criteria that take into account the patient’s history and clinical manifestations. Successful management of the disorder requires a multifaceted approach that involves education, optimal skin care practices, anti-inflammatory treatment with topical corticosteroids and/or topical calcineurin inhibitors, the management of pruritus, and the treatment of skin infections. Systemic immunosuppressive agents may also be used, but are generally reserved for severe flare-ups or more difficult-to-control disease. Topical corticosteroids are the first-line pharmacologic treatments for AD, and evidence suggests that these agents may also be beneficial for the prophylaxis of disease flare-ups. Although the prognosis for patients with AD is generally favourable, those patients with severe, widespread disease and concomitant atopic conditions, such as asthma and allergic rhinitis, are likely to experience poorer outcomes.

## Background

Atopic dermatitis (AD) is a chronic, highly pruritic (itchy) inflammatory skin disease, and is one of the most common skin disorders in children [1]. The disorder results in significant morbidity and adversely affects quality of life [2]. Not only are patients affected by the social stigma of a visible skin condition, but the intense itching characteristic of the disease often leads to skin trauma and significant sleep disturbances. In addition, management of the condition necessitates the frequent application of emollients (agents that soothe, moisturize and soften the skin) and topical medications, as well as physician visits. AD also poses a significant economic burden with an estimated annual cost in Canada of $1.4 billion [3].

Current evidence suggests that AD is a primary skin barrier defect that facilitates the development of other atopic conditions [4, 5]. In fact, AD is often the initial step in the “atopic march” (the sequential development of allergic disease manifestations during early childhood), which leads to asthma and/or allergic rhinitis in the majority of afflicted patients [6]. Early AD may also be a causative factor in the development of food allergy [7].

Newer insights into AD suggest that both structural abnormalities of the skin and immune dysregulation play important roles in the pathophysiology of the disease. Therefore, optimal management of AD requires a multifaceted approach aimed at healing and protecting the skin barrier and addressing the complex immunopathogenesis of the disease [8, 9]. This article provides an overview of current literature related to the epidemiology, pathophysiology, diagnosis, and appropriate management of AD.

## Pathophysiology

The pathogenesis of AD is not completely understood, however, the disorder appears to result from the complex interaction between defects in skin barrier function, immune dysregulation, and environmental and infectious agents [4, 5, 10]. Skin barrier abnormalities appear to be associated with mutations within or impaired expression of the filaggrin gene, which encodes a structural protein essential for skin barrier formation. The skin of individuals with AD has also been shown to be deficient in ceramides (lipid molecules) as well as antimicrobial peptides such as cathelicidins, which represent the first-line of defense against many infectious agents. These skin barrier abnormalities lead to transepidermal water loss (passage of water from inside the body through the epidermal layer of the skin to the surrounding atmosphere) and increased penetration of allergens and microbes into the skin. The infectious agent most often involved in AD is *Staphylococcus aureus* (*S. aureus*), which colonizes in approximately 90% of AD patients. Defective innate immune responses also appear to contribute to increased bacterial and viral infections in patients with AD. This interplay of factors leads to T cell responses in the skin (initially a predominantly T helper-2 [Th2] response and later a predominantly Th1 response) with resultant release of chemokines and proinflammatory cytokines (e.g., interleukin [IL]-4, IL-5 and tumour necrosis factor) that promote immunoglobulin E (IgE) production and systemic inflammatory responses, leading to pruritic inflammation of the skin.

## Epidemiology

The prevalence of AD has increased over the past 30 years. It is currently estimated that 10–20% of children and 1–3% of adults in developed countries are affected by the disorder [11]. AD often starts in early infancy; approximately 45% of all cases begin within the first 6 months of life, 60% during the first year, and 85% before 5 years of age. In fact, many neonates destined to develop AD already have measurably increased transepidermal water loss on their second day of life [12], and this finding is strongly predictive of future food allergy [13]. Fortunately, up to 70% of children with AD will go into clinical remission before adolescence [14, 15].

As mentioned earlier, children with AD are at high risk of developing food allergies, asthma and allergic rhinitis. Severe AD in infancy is a major risk factor for allergies to egg and peanut [7, 13, 16]. Results of a recent systematic review suggest that AD of increased severity and chronicity is particularly associated with food allergy, and that AD precedes the development of food allergy, suggesting a causal relationship [7]. Evidence also suggests that of those who develop AD before the age of 2, 50% will develop asthma during subsequent years. Furthermore, those children with AD who develop asthma and allergic rhinitis are more likely to have severe disease [17].

## Diagnosis

There are no specific diagnostic tests for AD. Diagnosis of the disorder is based on specific criteria that take into account the patient’s history and clinical manifestations. Although various diagnostic criteria for AD have been proposed and validated, the application of many of these criteria is time consuming and often necessitates invasive testing. Table 1 provides simplified criteria proposed by Williams et al. that are easy to use, do not require invasive testing, and have been shown to have a high sensitivity and specificity for the diagnosis of AD [18–21]. Using these criteria, the diagnosis of AD requires the presence of an itchy skin condition (or parental/caregiver reports of scratching or rubbing in a child) plus three or more minor criteria, which vary depending on the patient’s age.

**Table 1:** Diagnostic criteria for AD [18–20]

**Major criteria**
Patient must have
• An itchy skin condition (or parental/caregiver report of scratching or rubbing in a child)
**Minor criteria**
Plus three or more of the following minor criteria
*Older children/adults*
• History of itchiness in skin creases (e.g., folds of elbows, behind the knees, front of ankles, around the neck)
• Personal history of asthma or allergic rhinitis
• Personal history of general dry skin in the last year
• Visible flexural dermatitis (i.e., in the bends or folds of the skin at the elbow, knees, wrists, etc.)
• Onset under age 2 years
*Children < 4 years*^a^
• History of itching of the cheeks
• History of atopic disease in a first-degree relative
• Eczema of cheeks, forehead and outer limbs

^a^Early onset not always diagnostic in children under 4 years of age

**Fig. 1:**
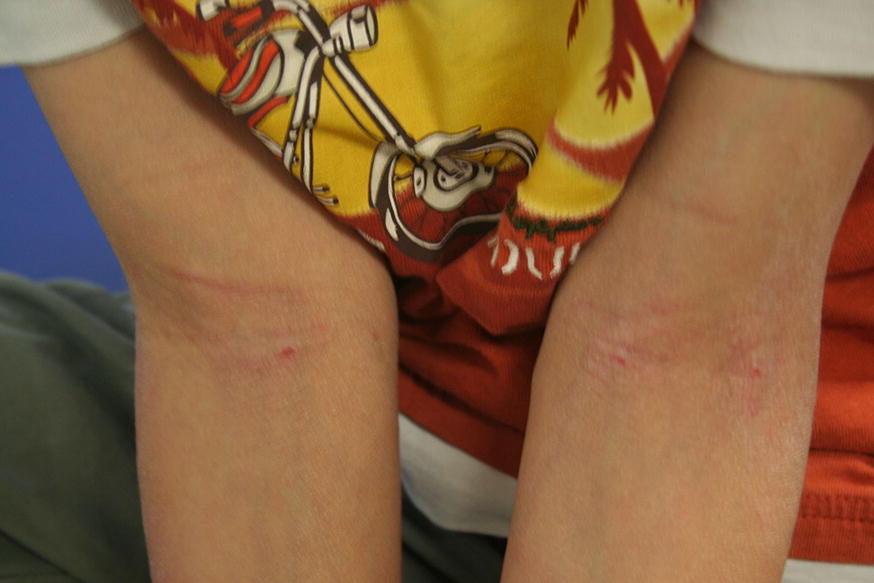
**AD of the flexural surfaces of the extremities**

**Fig. 2:**
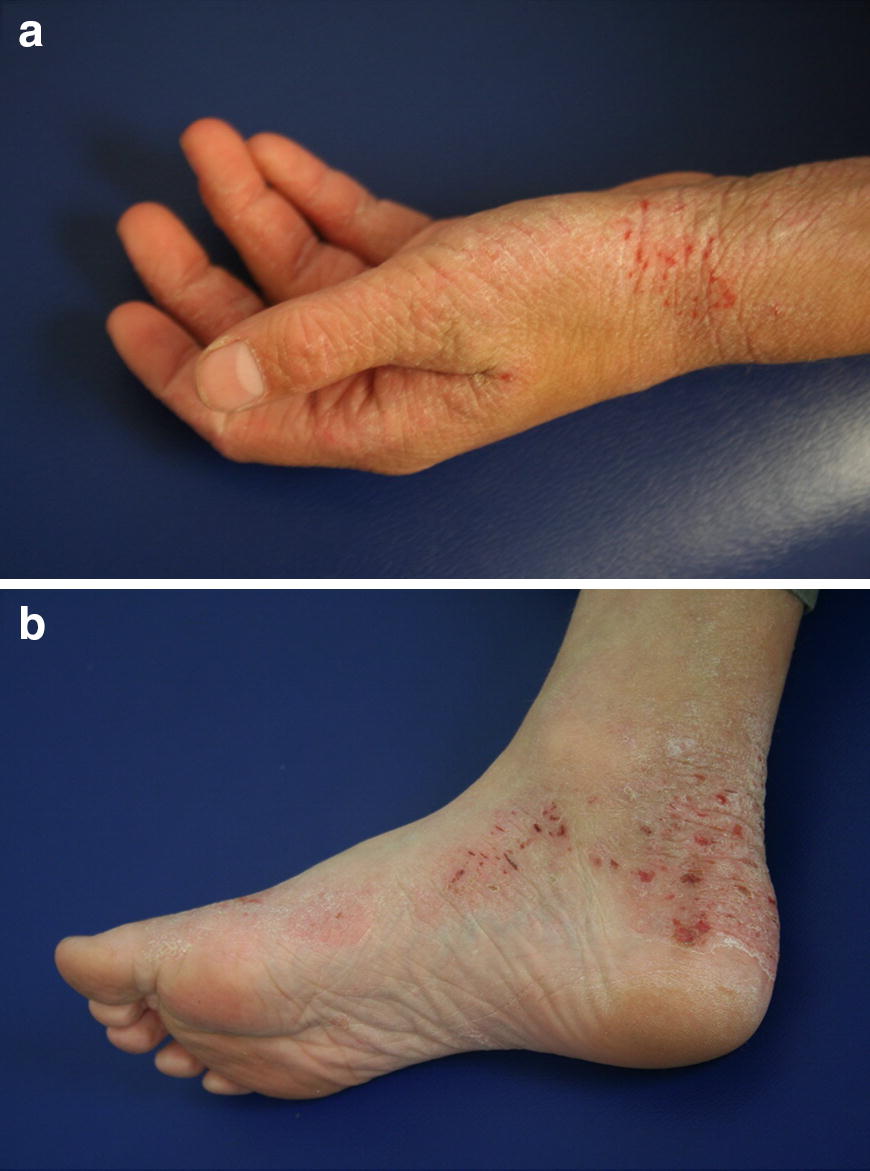
**AD of the hands (a) and feet (b)**

The clinical manifestations of AD vary with age (see Table 2). In infants, the scalp, face, neck, trunk and extensor (outer) surfaces of the extremities are generally affected, while the diaper area is usually spared. Children typically have involvement of the flexural surfaces of the extremities (i.e., fold/bend at the elbow and back of the knee), neck, wrists and ankles (see Fig. 1). In adolescence and adulthood, the flexural surfaces of the extremities, hands and feet are usually affected (see Fig. 2). Regardless of age, the itching associated with AD generally continues throughout the day and worsens at night, leading to sleep loss and substantial impairments in quality of life [2, 9].

**Table 2:** Clinical manifestations of AD

**Infants (0–2 years)**
• Extensor surfaces of extremities
• Face (forehead, cheeks, chin)
• Neck
• Scalp
• Trunk
**Childhood (2 years to puberty)**
• Flexural surfaces of extremities
• Neck
• Wrists, ankles
**Adolescence/adulthood**
• Flexural surfaces of extremities
• Hands, feet

It is sometimes difficult to differentiate AD from other skin conditions (e.g., seborrheic dermatitis, contact dermatitis, psoriasis, scabies); however, a family history of atopy and the distribution of lesions are helpful in making the diagnosis in many cases. Psoriasis, for example, usually affects the extensor rather than flexural surfaces, and often involves the fingernails, palms of the hands and soles of the feet. Seborrheic dermatitis typically involves the diaper area and scalp in infants, and the face in adults (e.g., sides of the nose, eyebrows, external ear canal). Furthermore, unlike AD, a family history of atopic disease is less common in patients with seborrheic or contact dermatitis. Scabies is generally associated with the presence of pustules on the palms, soles, genitalia and between the fingers. Other conditions that need to be considered in the differential diagnosis of AD are nutritional deficiencies, malignancies, and keratinization or immunodeficiency disorders that are associated with skin manifestations (see Table 3) [9].

**Table 3:** Common differential diagnosis of AD [9]

	Main age group affected	Frequency^a^	Characteristics and clinical features
**Other types of dermatitis**			
Seborrheic dermatitis	Infants	Common	Salmon-red greasy scaly lesions, often on the scalp (cradle cap) and napkin area; generally presents in the first 6 weeks of life; typically clears within weeks
	Adults	Common	Erythematous patches with yellow, white, or grayish scales in seborrheic areas, particularly the scalp, central face, and anterior chest
Nummular dermatitis	Children and adults	Common	Coin-shaped scaly patches, mostly on legs and buttocks; usually no itch
Irritant contact dermatitis	Children and adults	Common	Acute to chronic eczematous lesions, mostly confined to the site of exposure; history of locally applied irritants is a risk factor; might coexist with AD
Allergic contact dermatitis	Children and adults	Common	Eczematous rash with maximum expression at sites of direct exposure but might spread; history of locally applied irritants is a risk factor; might coexist with AD
Lichen simplex chronicus	Adults	Uncommon	One or more localised circumscribed lichenified plaques that result from repetitive scratching or rubbing because of intense itch
Asteatotic eczema	Adults	Common	Scaly, fissured patches of dermatitis overlying dry skin, most often on lower legs
**Infectious skin diseases**			
Dermatophyte infection	Children and adults	Common	One or more demarcated scaly plaques with central clearing and slightly raised reddened edge; variable itch
Impetigo	Children	Common	Demarcated erythematous patches with blisters or honey-yellow crusting
Scabies	Children	Common^b^	Itchy superficial burrows and pustules on palms and soles, between fingers, and on genitalia; might produce secondary eczematous changes
**Congenital immunodeficiencies**			
Hyper-IgE syndrome	Infants	Rare	Pustular and eczematous rashes within first weeks of life; staphylococcal infections of the skin, sinuses, and lungs; high serum IgE; eosinophilia
Wiskott-Aldrich syndrome	Infants	Very rare	Rash identical to that of AD, usually in first weeks of life in boys; microthrombocytopenia
Omenn syndrome	Infants	Very rare	Early-onset erythroderma, diffuse scaly rash, and chronic diarrhea
**Keratinization disorders**			
Ichthyosis vulgaris	Infants and adults	Uncommon	Dry skin with fine scaling, particularly on the lower abdomen and extensor areas; perifollicular skin roughening; palmar hyperlinearity; full form (i.e., 2 *FLG* mutations) is uncommon; often coexists with AD
Netherton syndrome	Infants and adults	Very rare	Eczematous lesions spread over the skin in a serpiginous linear pattern with double-edged scales; hair shaft anomalies (bamboo hair); increased IgE; eosinophilia
**Nutritional deficiency**			
Zinc deficiency	Children	Uncommon	Erythematous scaly patches and plaques most often around the mouth and anus; rare congenital form accompanied by diarrhea and alopecia
**Neoplastic disease**			
Cutaneous T-cell lymphoma	Adults	Uncommon	Erythematous pink-brown macules and plaques with a fine scale; poorly responsive to topical steroids; variable itch (in early stages)

Adapted from Weidinger and Novak [9]

*FLG* filaggrin, *AD* atopic dermatitis

^a^Common = roughly 1 in 10 to 1 in 100; uncommon = roughly 1 in 100 to 1 in 1000; rare = roughly 1 in 1000 to 1 in 10,000; very rare = less than 1 in 10,000

^b^Especially in developing countries

## Allergy assessment

The exact role of foods and aeroallergens in the pathogenesis and exacerbation of AD is controversial. Although most patients with AD demonstrate specific IgE antibodies to foods and/or aeroallergens on skin prick testing (SPT) and measurements of serum-specific IgE levels, their clinical significance remains unclear [17, 22]. In other words, while a positive SPT or serum-specific IgE test indicates sensitization to a particular allergen, this does not prove clinical hypersensitivity or causation.

In clinical studies, approximately 35% of children with moderate-to-severe AD have been found to have contributory food allergies [22]. In general, the younger the patient and the more severe the AD, the more likely it is that specific food allergens may exacerbate the disease, however this is usually apparent in the clinical history. In contrast, food allergies appear to have little, if any, role in adult AD [17].

Random testing or screening to food allergens is not recommended as this may lead to unnecessary and inappropriate dietary restrictions in patients with AD. The positive predictive value of screening panels of food allergens in such cases is as low as 2%, and these screening panels are associated with significant health care utilization [23]. Therefore, the decision to perform allergy testing to foods should be based on whether or not the patient’s history is highly suggestive of food allergies [22]. Note that children with food-triggered AD are often instructed to begin strict elimination diets of the offending food. However, recent evidence suggests that these elimination diets should be prescribed with caution as they can inadvertently lead to loss of tolerance of foods and increase the risk of immediate, IgE-mediated food reactions [24].

Exposure to aeroallergens such as house dust mites, animal dander, pollen and moulds can exacerbate AD in some patients. In these cases, identification of sensitization by SPT may be useful. If sensitization is established, and the history suggests a causative role in worsening AD, then specific avoidance measures should be considered since removal of the allergen from the patient’s environment may improve the symptoms of AD. Atopy patch testing is still considered investigational in patients with AD because there are no standardized methods of application or test interpretation. However, patch tests may be useful for excluding a diagnosis of concurrent contact dermatitis [17].

## Prevention

Although there are currently no established primary prevention strategies for AD, recent trials have demonstrated the effectiveness of early, consistent application of emollients for infants at increased risk. This simple and cost-effective approach has resulted in a 30–50% reduction in the diagnosis of AD at 6 months [25–27]. By reducing AD, this intervention may have the potential to prevent food allergy.

## Treatment

The treatment of AD should be directed at restoring the skin barrier, which includes hydrating and repairing the skin, limiting itching, and decreasing inflammation when necessary. Therefore, the successful management of AD requires a multifaceted approach that involves patient and caregiver education, optimal skin care practices, anti-inflammatory treatment with topical corticosteroids (first-line) and/or topical calcineurin inhibitors (TCIs), and the treatment of skin infections [1, 8, 9, 17]. Systemic immunosuppressive agents may also be considered in severe cases that cannot be controlled with appropriate skin care and topical therapy. Although first-generation antihistamines are not routinely recommended for the management of AD due to their sedative and impairing side effects, short-term use of these agents may be helpful in those individuals experiencing severe flares of AD, particularly if these flares are associated with significant sleep disturbances.

**Fig. 3:**
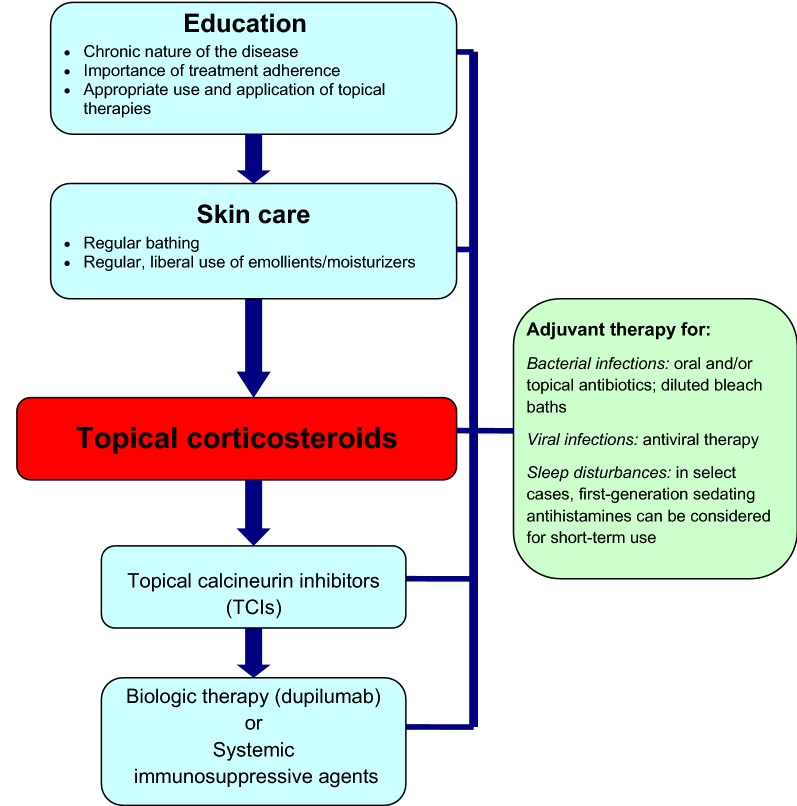
**A simplified, stepwise algorithm for the treatment of AD**

A simplified, stepwise algorithm for the treatment of AD is provided in Fig. 3. Physicians should monitor patient progress and disease course regularly and evaluate the efficacy and tolerability of therapy. Follow-up evaluations should include an assessment of medication use (e.g., type, quantity applied, refills made, etc.), which allows the physician to gauge compliance and medication risks.

### Education

For optimal disease management, patients and/or their caregivers should be educated about the chronic nature of the disease, the need for continued adherence to proper skin care practices, and the appropriate use and application of topical therapies. Poor treatment outcomes are often related to poor adherence, especially to topical therapies, resulting from irrational fears about adverse effects and insufficient information [9]. Time spent addressing these fears and educating patients and caregivers has been shown to have a positive effect on disease outcomes. Patients should also be provided with written instructions/information on appropriate medication use, skin care and flare management to reinforce learning.

### Skin care principles

A key feature of AD management is appropriate daily skin care. Although the frequency of bathing is somewhat controversial, most experts suggest daily bathing [28]. Bathing once or twice daily (depending on the severity of AD) in warm water for 10–15 min is recommended to help hydrate and cleanse the skin, assist in the debridement of infected skin, and improve the penetration of topical therapies. Moisturizing cleansers are recommended while highly fragranced soaps should be avoided as they may irritate the skin. After bathing, the patient’s skin should be patted dry with a towel (so it remains slightly wet) and moisturizers/emollients should be applied liberally to help prevent moisture loss and drying of the skin. Note that creams and ointments are more effective for moisturizing the skin than lotions. Experts recommend that patients purchase inexpensive brands of creams or ointments that are available in large containers/jars.

### Topical corticosteroids

Topical corticosteroids are the first-line pharmacologic treatments for AD. These agents effectively control atopic flares through their anti-inflammatory, antiproliferative, and immunosuppressive actions. Numerous topical corticosteroids are available in Canada, ranging from low to high potency, and most of these agents are available in varying concentrations, preparations and doses (see Table 4). Topical corticosteroids are applied to the red, itchy or inflamed areas on the skin before the use of emollients. Some patients have inadvertently reversed the order, which significantly reduces the benefits of the topical corticosteroid.

**Table 4:** Potency of common topical corticosteroid therapies

**Very potent** • Betamethasone dipropionate (Diprolene) • Clobetasol propionate 0.05% (Dermovate) • Halobetasol propionate (Ultravate) • Halcinonide 0.1% (Halog) **Potent** • Amcinonide 0.1% (Cyclocort) • Betamethasone valerate 0.1% (Betaderm, Celestoderm, Prevex) • Desoximetasone 0.25% (Desoxi, Topicort) • Diflucortolone valerate 0.1% (Nerisone) • Fluocinolone acetonide 0.25% (Derma, Fluoderm, Synalar) • Fluocinonide 0.05% (Lidemol, Lidex, Tiamol, Topsyn) • Fluticasone propionate (Cutivate) • Mometasone furoate 0.1% (Elocon)	**Moderately potent** • Betamethasone valerate 0.05% (Betnovate, Celestoderm) • Clobetasone butyrate 0.05% (Eumovate) • Hydrocortisone valerate 0.2% (Westcort, HydroVal) • Prednicarbate 0.1% (Dermatop) • Triamcinolone acetonide 0.1% (Aristocort R, Kenalog, Traiderm) **Mild** • Desonide (Desocort) • Hydrocortisone acetate 0.5–2% (Cortef, Hyderm, Cortate, Cortoderm)

There is limited clinical trial data to assist in choosing a corticosteroid. Ointment preparations are generally preferred over creams as they provide more uniform coverage and penetration. Also, the least potent preparation required to control AD (particularly in sensitive areas such as the face, neck, groin and underarms) should be utilized. Often, a low-potency preparation, such as hydrocortisone acetate 1% or equivalent, is used for the face.

When used appropriately, topical corticosteroids are extremely safe and effective. Possible local side effects of long-term topical corticosteroid use include striae (stretch marks), petechiae (small red/purple spots), telangiectasia (small, dilated blood vessels on the surface of the skin), skin thinning, atrophy and acne; however, these effects are uncommon with low or moderate potency preparations. Systemic side effects with topical corticosteroid use are rare, and are usually associated with higher-potency preparations being applied to a large body surface area.

Evidence also suggests that topical corticosteroids may be beneficial for the prophylaxis of AD flares. Studies have found that, after AD is stabilized, the addition of twice-weekly topical anti-inflammatories to maintenance treatment with emollients significantly reduces the risk of relapses in both pediatric and adult subjects [29].

### Topical calcineurin inhibitors (TCIs)

TCIs are immunosuppressant agents that have also been shown to be safe and effective for the treatment of AD [9, 30, 31], as well as the prophylaxis of AD flares [29]. Two TCIs—pimecrolimus (Elidel) and tacrolimus (Protopic)—are currently approved in Canada for the second-line, intermittent treatment of immunocompetent patients 2 years of age and older with moderate-to-severe AD. Given the high costs of these agents, they are generally reserved for patients with persistent disease and/or frequent flares that would require continuous topical corticosteroid treatment, or in patients severely affected in sensitive skin areas (e.g., around the eyes, face, neck and genitals) where systemic absorption and the risk of skin atrophy with topical corticosteroids are of particular concern.

The most common local adverse effects of TCIs are skin burning and irritation, which often improve with continued use. Although both Health Canada and the Food and Drug Administration (FDA) have recommended caution when prescribing TCIs due to rare reports of skin malignancy and lymphoma in patients using these agents, the Canadian Society of Allergy and Clinical Immunology (CSACI) released a position statement highlighting that, to date, there has been no published evidence showing that TCIs clearly predispose to malignancy. The CSACI concluded that TCIs are effective treatments for AD, and that the benefits of their use in the appropriately selected patient population outweighs the theoretical risk of increased malignancy [32].

### Treatment of skin infections

The skin of patients with AD is often heavily colonized with *S. aureus*, even at uninvolved sites. Short-term topical and/or oral antibiotic therapy is recommended when an overt secondary bacterial infection is present. Appropriate systemic antibiotics are indicated for widespread secondary infection, and first- or second-generation cephalosporins or anti-staphylococcal penicillins for 7–10 days are usually effective in managing the infection. Because erythromycin-resistant organisms are common in patients with AD, macrolides are less useful alternatives [17].

Patients with AD are also prone to recurrent viral infections. Eczema herpeticum (a severe disseminated herpes infection that generally occurs at sites of skin damage; also known as Kaposi’s varicelliform eruption) is a serious risk in patients with widespread AD and may be easily misdiagnosed as a bacterial superinfection. Patients with this condition will require systemic antiviral treatment with acyclovir or other antiviral agents [17]. Molluscum contagiosum (a common viral cutaneous infection caused by a poxvirus of the *Molluscipox* genus) is often seen in children with AD. Although the infection is usually self-limited, the lesions often resolve slowly and tend to spread in patients with AD [9]. Severe, persistent molluscum contagiosum infection may require laser and/or antiviral therapy.

Diluted bleach baths are also recommended to help reduce the number of *S. aureus* skin infections, and the need for systemic antibiotics in patients with heavily colonized skin. Diluted bleach baths involve soaking the patient for approximately 10 min in a tub full of lukewarm water that is mixed with one-quarter to one-half cup (60–120 mL) of chlorine bleach (this concentration is similar to the amount of chlorine in a pool). The patient is then thoroughly rinsed with fresh water, and a moisturizer or emollient is applied immediately to prevent dehydration and dryness [1]. Twice-weekly diluted bleach baths for a period of 3 months have been recommended by some authors [33].

### Systemic immunosuppressive agents

Short-term treatment with systemic immunosuppressive agents, such as cyclosporine, azathioprine and methotrexate, has been shown to be effective in patients failing topical treatment and, therefore, these agents are often recommended for severe, refractory AD [8, 9]. However, it is important to note that discontinuation of cyclosporine often leads to rapid disease relapse. Also, patients treated with these immunosuppressive agents should be monitored for potential adverse effects, such as kidney or liver function impairment with cyclosporine, and myelosuppression with azathioprine. Therefore, referral to a specialist is warranted for AD patients who may be candidates for systemic immunosuppressive therapy.

Systemic corticosteroids have an unfavourable risk–benefit profile, and there is currently insufficient evidence supporting their use in AD. Therefore, these agents should be reserved for exceptional cases, and prolonged use should be avoided given their potential for serious adverse events [9].

### Antihistamines

Although first-generation antihistamines (e.g., hydroxyzine, diphenhydramine, chlorpheniramine) do not directly affect the itching associated with AD, the sedative effects of these agents have been found to help improve sleep in patients with AD [1, 17]. However, these agents have been found to reduce rapid eye movement (REM)-sleep, impair learning and reduce work efficiency [34] and, therefore, are not routinely recommended for patients with AD. They may be considered for the short-term adjuvant treatment of patients experiencing severe AD flare-ups who have difficulty sleeping or who scratch regularly while sleeping. Long-term and/or daytime use of first-generation antihistamines should be avoided given their sedative properties. Non-sedating second-generation antihistamines appear to provide modest benefit in AD patients with allergic triggers [1, 17] and, hence, a therapeutic trial of these agents may be considered in certain clinical situations.

### Other therapies

Ultraviolet (UV) phototherapy may be beneficial for the treatment of AD in adults. However, the long-term toxicity of UV therapy is still unknown. Allergen-specific immunotherapy may also be effective in select patients with AD that is associated with aeroallergen sensitization (see *Allergen-Specific Immunotherapy* article in this supplement) [35–37].

Although some studies have found wet-wrap therapy (the application of wet bandages over AD lesions after applying emollients and/or topical corticosteroids) to be effective for the treatment of AD, others have questioned its effectiveness and emphasize the potential for associated complications such as local infections [9]. A recent systematic review of trials comparing wet wrap therapy to conventional topical corticosteroid treatment in patients with AD found no good quality evidence to suggest that wet wraps are superior to conventional topical therapies [38].

A number of biologic agents targeting the immune pathways involved in AD are under investigation and may represent promising future therapies for the condition. Recently, dupilumab (a fully human monoclonal antibody directed against the alpha subunit of the interleukin-4 [IL-4] receptor) has been approved in Canada for the treatment of moderate-to-severe AD that is not adequately controlled with topical therapies or when these therapies are not advisable. Two phase 3 trials found dupilumab to significantly improve symptoms and quality of life in patients with AD compared to placebo [39].

## Prognosis

The prognosis for patients with AD is generally favourable, with most children outgrowing the condition by early adolescence. However, patients with severe, widespread disease and concomitant atopic conditions, such as asthma and allergic rhinitis, are likely to experience poorer outcomes [15].

## Conclusions

Atopic dermatitis is a common, chronic skin disease that starts early in life and can adversely impact the quality of life of patients and their caregivers. Optimal skin care practices and topical corticosteroids remain the cornerstone of therapy for the disease. TCIs have been shown to provide an effective, second-line alternative to topical corticosteroids in appropriate patients prone to frequent flare-ups. Systemic immunosuppressive agents may also be considered in severe cases that cannot be controlled with appropriate skin care and topical therapies. Allergy testing to foods and aeroallergens may be considered based on patient history and/or in patients exhibiting a poor response to optimal skin care practices and appropriate pharmacological therapy. A number of biologics, such as dupilumab, are being investigated in AD and may represent promising future options for management of this debilitating skin disorder.

## Key take-home messages

- AD is the most common skin disorder in children, and significantly impacts quality of life.
- The diagnosis of AD is based on specific diagnostic criteria that take into account the patient’s history and clinical manifestations.
- Random or screening allergy tests to foods are not recommended. Allergy testing using SPTs or serum-specific IgE measurements may be useful for identifying triggers of AD if the patient’s history is suggestive of allergies to foods or other environmental factors.
- Early, consistent application of emollients may help prevent AD in infants at increased risk.
- Optimal skin care practices and topical corticosteroids are the mainstay of therapy for AD.
- TCIs are a second-line alternative to topical corticosteroids.
- The skin of most patients with AD is heavily colonized with *S. aureus*; therefore, topical and/or systemic antibiotic therapy may be required for overt infections.
- Specialist referral may be helpful for severe flare-ups or more difficult-to-control disease. In these cases, systemic immunosuppressive agents may be utilized.

## Abbreviations

AD: atopic dermatitis
Th2: T helper cell type-2
IL: interleukin
IgE: immunoglobulin E
TCIs: topical calcineurin inhibitors
SPT: skin prick testing
CSACI: Canadian Society of Allergy and Clinical Immunology
FDA: Food and Drug Administration

## References

[1] Krakowski AC, Eichenfield LF, Dohil MA (2008). Management of atopic dermatitis in the pediatric population. Pediatrics.

[2] McKenna SP, Doward LC (2008). Quality of life of children with atopic dermatitis and their families. Curr Opin Allergy Clin Immunol.

[3] Barbeau M, Bpharm HL (2006). Burden of atopic dermatitis in Canada. Int J Dermatol.

[4] Egawa G, Kabashima K (2016). Multifactorial skin barrier deficiency and atopic dermatitis: essential topics to prevent the atopic march. J Allergy Clin Immunol.

[5] Nomura T, Kabashima K (2016). Advances in atopic dermatitis in 2015. J Allergy Clin Immunol.

[6] Spergel JM, Paller AS (2003). Atopic dermatitis and the atopic march. J Allergy Clin Immunol.

[7] Tsakok T, Marrs T, Mohsin M, Baron S, du Toit G, Till S, Flohr C (2016). Does atopic dermatitis cause food allergy? A systematic review. J Allergy Clin Immunol.

[8] Lee JH, Son SW, Cho SH (2016). A comprehensive review of the treatment of atopic eczema. Allergy Asthma Immunol Res.

[9] Weidinger S, Novak N (2016). Atopic dermatitis. Lancet.

[10] Fonacier LS, Dreskin SC, Leung DYM (2010). Allergic skin diseases. J Allergy Clin Immunol.

[11] Larsen FS, Hanifin JM (2002). Epidemiology of atopic dermatitis. Immunol Allergy Clin North Am.

[12] Kelleher M, Dunn-Galvin A, Hourihane JO, Murray D, Campbell LE, McLean WH, Irvine AD (2015). Skin barrier dysfunction measured by transepidermal water loss at 2 days and 2 months predates and predicts atopic dermatitis at 1 year. J Allergy Clin Immunol.

[13] Kelleher MM, Dunn-Galvin A, Gray C, Murray DM, Kiely M, Kenny L, McLean WH, Irvine AD, Hourihane JO (2016). Skin barrier impairment at birth predicts food allergy at 2 years of age. J Allergy Clin Immunol.

[14] Pyun BY (2015). Natural history and risk factors of atopic dermatitis in children. Allergy Asthma Immunol Res.

[15] Bieber T (2008). Mechanisms of disease: atopic dermatitis. N Engl J Med.

[16] Brough HA, Liu AH, Sicherer S, Makinson K, Douiri A, Brown SJ, Stephens AC, Irwin McLean WH, Turcanu V, Wood RA (2015). Atopic dermatitis increases the effect of exposure to peanut antigen in dust on peanut sensitization and likely peanut allergy. J Allergy Clin Immunol.

[17] Akdis CA, Akdis M, Bieber T, Bindslev-Jensen C, Boguniewicz M, Eigenmann P, Hamid Q, Kapp A, Leung DY, Lipozencic J, European Academy of Allergology and Clinical Immunology/American Academy of Allergy, Asthma and Immunology (2006). Diagnosis and treatment of atopic dermatitis in children and adults: European Academy of Allergology and Clinical Immunology/American Academy of Allergy, Asthma and Immunology/PRACTALL Consensus Report. J Allergy Clin Immunol.

[18] Williams HC, Burney PG, Hay RJ, Archer CB, Shipley MJ, Hunter JJ, Bingham EA, Finlay AY, Pembroke AC, Graham-Brown RA (1994). The U.K. Working Party’s diagnostic criteria for atopic dermatitis. I. Derivation of a minimum set of discriminators for atopic dermatitis. Br J Dermatol.

[19] Williams HC, Burney PG, Strachan D, Hay RJ (1994). The U.K. Working Party’s diagnostic criteria for atopic dermatitis. II. Observer variation of clinical diagnosis and signs of atopic dermatitis. Br J Dermatol.

[20] Williams HC, Burney PG, Pembroke AC (1994). The U.K. Working Party’s diagnostic criteria for atopic dermatitis. III. Independent hospital validation. Br J Dermatol.

[21] Gu H, Chen XS, Chen K, Yan Y, Jing H, Chen XQ, Shao CG, Ye GY (2001). Evaluation of diagnostic criteria for atopic dermatitis: validity of the criteria of Williams et al in a hospital-based setting. Br J Dermatol.

[22] Kim JS (2008). Pediatric atopic dermatitis: the importance of food allergens. Semin Cutan Med Surg.

[23] Bird JA, Crain M, Varshney P (2015). Food allergen panel testing often results in misdiagnosis of food allergy. J Pediatr.

[24] Chang A, Robison R, Cai M, Singh AM (2016). Natural history of food-triggered atopic dermatitis and development of immediate reactions in children. J Allergy Clin Immunol Pract.

[25] Simpson EL, Chalmers JR, Hanifin JM, Thomas KS, Cork MJ, McLean WH, Brown SJ, Chen Z, Chen Y, Williams HC (2014). Emollient enhancement of the skin barrier from birth offers effective atopic dermatitis prevention. J Allergy Clin Immunol.

[26] Horimukai K, Morita K, Narita M, Kondo M, Kitazawa H, Nozaki M, Shigematsu Y, Yoshida K, Niizeki H, Motomura K (2014). Application of moisturizer to neonates prevents development of atopic dermatitis. J Allergy Clin Immunol.

[27] Xu S, Immaneni S, Hazen GB, Silverberg JI, Paller AS, Lio PA (2017). Cost-effectiveness of prophylactic moisturization for atopic dermatitis. JAMA Pediatr.

[28] Gittler JK, Wang JF, Orlow SJ (2017). Bathing and associated treatments in atopic dermatitis. Am J Clin Dermatol.

[29] Schmitt J, von Kobyletzki L, Svensson A, Apfelbacher C (2011). Efficacy and tolerability of proactive treatment with topical corticosteroids and calcineurin inhibitors for atopic eczema: systematic review and meta-analysis of randomized controlled trials. Br J Dermatol.

[30] Broeders JA, Ahmed Ali U, Fischer G (2016). Systematic review and meta-analysis of randomized clinical trials (RCTs) comparing topical calcineurin inhibitors with topical corticosteroids for atopic dermatitis: a 15-year experience. J Am Acad Dermatol.

[31] Siegfried EC, Jaworski JC, Kaiser JD, Hebert AA (2016). Systematic review of published trials: long-term safety of topical corticosteroids and topical calcineurin inhibitors in pediatric patients with atopic dermatitis. BMC Pediatr.

[32] Segal AO, Ellis AK, Kim HL (2013). CSACI position statement: safety of topical calcineurin inhibitors in the management of atopic dermatitis in children and adults. Allergy Asthma Clin Immunol.

[33] Huang JT, Abrams M, Tlougan B, Rademaker A, Paller AS (2009). Treatment of *Staphylococcus aureus* colonization in atopic dermatitis decreases disease severity. Pediatrics.

[34] Church MK, Maurer M, Simons FE, Bindslev-Jensen C, van Cauwenberge P, Bousquet J, Holgate ST, Zuberbier T, Global Allergy and Asthma European Network (2010). Risk of first-generation H(1)-antihistamines: a GA(2)LEN position paper. Allergy.

[35] Canadian Society of Allergy and Clinical Immunology. Immunotherapy manual. 2016. http://csaci.ca/wp-content/uploads/2017/12/IT-Manual-2016-5-July-2017-rev.pdf. Accessed 12 July 2018.

[36] Cox L, Nelson H, Lockey R, Calabria C, Chacko T, Finegold I, Nelson M, Weber R, Bernstein DI, Blessing-Moore J, Khan DA, Lang DM, Nicklas RA, Oppenheimer J, Portnoy JM, Randolph C, Schuller DE, Spector SL, Tilles S, Wallace D (2011). Allergen immunotherapy: a practice parameter third update. J Allergy Clin Immunol.

[37] Bae JM, Choi YY, Park CO, Chung KY, Lee KH (2013). Efficacy of allergen-specific immunotherapy for atopic dermatitis: a systematic review and meta-analysis of randomized controlled trials. J Allergy Clin Immunol.

[38] González-López G, Ceballos-Rodríguez RM, González-López JJ, Feito Rodríguez M, Herranz-Pinto P (2017). Efficacy and safety of wet wrap therapy for patients with atopic dermatitis: a systematic review and meta-analysis. Br J Dermatol.

[39] Simpson EL, Bieber T, Guttman-Yassky E, Beck LA, Blauvelt A, Cork MJ, Silverberg JI, Deleuran M, Kataoka Y, Lacour JP, Kingo K, Worm M, Poulin Y, Wollenberg A, Soo Y, Graham NM, Pirozzi G, Akinlade B, Staudinger H, Mastey V, Eckert L, Gadkari A, Stahl N, Yancopoulos GD (2016). Ardeleanu M; SOLO 1 and SOLO 2 Investigators. Two phase 3 trials of dupilumab versus placebo in atopic dermatitis. N Engl J Med.

